# Evaluation of the benefit of thermal spa therapy in plaque psoriasis: the PSOTHERMES randomized clinical trial

**DOI:** 10.1007/s00484-022-02273-7

**Published:** 2022-03-26

**Authors:** Marie Beylot-Barry, Emmanuel Mahé, Carole Rolland, Maud Amy de la Bretèque, Claire Eychenne, Julie Charles, Catherine Payen, Laurent Machet, Céline Vermorel, Alison Foote, Christian Roques, Jean-Luc Bosson

**Affiliations:** 1 Department of Dermatology, University Hospital of Bordeaux, Bordeaux, France; 2 French Psoriasis Research Group (GrPSO) of the French Society of Dermatology, Paris, France; 3 Department of Dermatology, Victor Dupouy Hospital, Argenteuil, France; 4 CNRS TIMC-IMAG Laboratory, University Grenoble Alpes, Grenoble, France; 5 Department of Dermatology, Grenoble Alpes University Hospital and INSERM U1209 University Grenoble Alpes, Grenoble, France; 6 Private Practice, Place Louis Jouvet, Grenoble, France; 7 Department of Dermatology, University Hospital of Tours, Tours, France; 8 Research Division, Grenoble Alpes University Hospital, Grenoble, France; 9 French Association for Thermal Research (AFRETH), Paris, France

**Keywords:** Thermal spa, Balneotherapy, Psoriasis, Quality of life, Dermatology Life Quality Index

## Abstract

Spa therapy is considered an add-on treatment for psoriasis, but without any objective evaluation in the absence of randomized trials. This multicenter, open-label, randomized trial compared immediate spa therapy versus a control group having usual treatments until study assessments at 4.5 months. Spa therapy was proposed in five French spa resorts with standardized programs. Inclusion criteria were adults with plaque psoriasis, Dermatology Life Quality Index (DLQI) > 10, and stable medical treatment in the last 6 months. The main objective was DLQI ≤ 10 at 4.5 months after inclusion. VQ-Dermato and EQ5D-3L also assessed quality of life (QoL), Perceived Stress Scale (PSS) stress, and visual analogue scales (VAS) pain and pruritus. Between January 2015 and November 2018, 128 patients were randomized to either immediate spa therapy (64) (within 34 days, median) or usual treatments (61) until assessment at 4.5 months. Most were first-time spa users (71.2%). Mean DLQI and Psoriasis Area and Severity Index at inclusion were 16.7 and 10.5, respectively. Immediate spa therapy patients achieved the primary objective for 66.1% [95% CI 52.6% > 77.9%] vs 41.4% [95% CI 28.6% > 55.1%] control group patients (*p* = 0.007). VQ-Dermato scores and pruritus VAS significantly improved. Outcomes at 12-month follow-up of the immediate spa therapy group showed persistent improvement of DLQI, VQ-Dermato, and pruritus. This randomized controlled trial demonstrated that a cure of spa therapy improves QoL and alleviates certain symptoms of psoriasis, in short and long terms. This justifies its integration in the therapeutic strategies for psoriasis. Trial registration number: ClinicalTrials.gov Identifier: NCT02098213.

**Supplementary Information:**

The online version contains supplementary material available at 10.1007/s00484-022-02273-7.

## Introduction

Psoriasis is a chronic inflammatory skin disease, affecting 2–3% of the general population. This disease is challenging due to its chronicity, and has a negative effect on quality of life (QoL) and a high prevalence of comorbidities (Lebwohl et al. 2014). In recent years, several therapeutic advances have improved the management of moderate to severe psoriasis, but some of these treatments may have side effects. The choice of treatment depends on the patient’s and the disease’s characteristics (sex, age, comorbidities, extent of lesions, treatment history; Nast et al. 2020). In a single patient, it may be necessary to use several different lines of treatment particularly because psoriasis is a lifelong disease. Moreover, in less severely affected patients, who make up the majority of the psoriasis population, topical drugs are the first-line treatment, but can be burdensome or difficult for patients to use and they may be associated with poor adherence in clinical practice (Caldarola et al. 2017).

Natural thermal waters have been used for their healing or curative properties for centuries (Kazandjieva et al. 2008; Cacciapuoti et al. 2020). Good quality studies suggest a clinical benefit of medical hydrology for several chronic pathologies, such as osteoarthritis and fibromyalgia (Antonelli et al. 2021), but few studies have evaluated specific disease-orientated hydrotherapy in a spa setting. Spa therapy, as practiced in French and other European thermal spa resorts, is a complex therapeutic intervention associating natural thermal mineral water balneotherapy (hydromassage baths, body jet showers, water affusion massages, and sometimes mineral-rich mud applications) with physiotherapy (such as supervised collective exercises in warm mineral water pools), well-being care, relaxation, and patient education (sometimes organized in specific programs).

In psoriasis, skin disease-orientated spa therapy is considered a safe complementary treatment and not an alternative to all other treatments (Matz et al. 2003; Huang et al. 2018). A course of spa therapy should allow a break before resorting to other systemic therapy. The benefit of spa therapy in a thermal resort has several potential facets: naturally hot mineral waters from hot springs (thermal hydrotherapy); codified treatments and exercises administered or supervised by trained spa therapy specialists; and a relaxing calm environment minimizing everyday stress. Thus, an improvement in QoL might be expected (Kazandjieva et al. 2008; Cacciapuoti et al. 2020; Matz et al. 2003). The thermal waters themselves vary in chemical composition, hydrogeologic origin, and temperature. They are thought to decrease skin inflammation (based on in vitro studies) and have an anti-pruritic effect (Kazandjieva et al. 2008; Cacciapuoti et al. 2020). Indeed, much of the literature in this field focuses on the effect of mineral water from one or other particular thermal source, on psoriasis (Tsoureli-Nikita et al. 2002) (Golusin et al. 2015). One study specifically investigated the effect of balneotherapy in selenium-rich mineral water on the skin microbiome of patients with psoriasis (Martin et al. 2015). Another suggested that the repeated application of arsenical-ferruginous spa water had a beneficial effect on psoriatic lesions (Borroni et al. 2013).

However, spa therapy in dermatology still suffers from lack of large-scale evaluation and especially objective assessment using reliable methodologies that limit bias. The treatment may consist of combinations of balneotherapy with heliotherapy or phototherapy, but only a few recent studies have attempted to assess the value and position the relative benefit of each therapeutic element, comparing balneotherapy associated with ultraviolet B (UVB) versus UVB only or versus balneotherapy only (Brockow et al. 2007; Léauté-Labrèze et al. 2001; Tabolli et al. 2009). These studies, in which assessments were based on the Psoriasis Area and Severity Index (PASI), suggested the superiority of the association; spa therapy enhancing the UVB effect, but either did not allow to demonstrate the positive impact of spa therapy alone (Brockow et al. 2007; Léauté-Labrèze et al. 2001) or only showed a short-term improvement that was mostly lost after 3 to 4 months (Peroni et al. 2008).

Therefore, to our knowledge, there are no randomized controlled multicenter clinical trials evaluating spa therapy alone for psoriasis.

Our aim was to perform a multicenter, controlled, parallel group, open-label randomized trial to assess the early and long-term benefit(s) of a 3-week course of dermatology-orientated spa therapy for patients with plaque psoriasis since at least 1 year. We focused on QoL, but also looked at other criteria such as pain, itching, and psoriasis severity.

## Methods

### Study design

This study, named “PSOTHERMES” (trial registration: NCT02098213), was a multicenter, controlled, parallel group, open-label randomized trial, with an immediate intervention—delayed intervention methodology. Patients with plaque psoriasis were randomized to the intervention group (spa treatment within 45 days of inclusion), or to the control group (spa treatment starting at least 4.5 months after the inclusion visit and after assessment of the primary endpoint).

The study was conducted in France between January 2015 and November 2018. A French ethics committee (CPP “Sud-Ouest et Outre Mer III” 2013) approved the study and informed written consent was obtained from all patients.

The 15 investigators involved in inclusion and assessment procedures were dermatologists in hospital or private practice, independent of the spa therapy centers, who had been recruited mainly through the French Psoriasis Research Group (GRPso).

### Study population, inclusion criteria, and assessment procedures

Inclusion criteria were as follows: patients over 18 years old, plaque psoriasis of more than 1-year duration, diagnosed by a dermatologist, Dermatology Life Quality Index (DLQI) score > 10, and stable treatment in the last 6 months. The patient had to be available for 3-week course of spa therapy within 45 days of inclusion and 4.5 months after inclusion according to randomization, and to attend 4 follow-up visits over 12 months.

The main non-inclusion criteria were phototherapy in the last 3 months; guttate, pustular or erythrodermic psoriasis, or isolated nail psoriasis; any contraindication to hydrotherapy (immune deficiency, progressive heart disease, progressive neoplasia, a contagious infectious disease, unhealed skin lesions); foreseeable intolerance of spa therapy (intolerance to heat, baths, swimming pools, etc.); pregnancy or breast feeding; psychiatric illness that would preclude study compliance; and recent spa therapy in the current spa resort season. All ongoing treatments were permitted by the protocol.

We recruited potential participants among the investigators’ patients, and also through the press, social networks, and the French Psoriasis Association. A first phase of screening was carried out by telephone by the coordination center to provide information about the study and to verify the individual’s eligibility. Randomization was centralized and electronic, through the e-CRF website.

For each participant, the study lasted 12 months, with clinical evaluations at the inclusion visit, at 4.5, 6, 9, and 12 months. At each visit, the data from a clinical examination and ongoing treatments were recorded and the participant filled in several questionnaires—DLQI (Basra et al. 2008), EuroQol, VQ-Dermato (Grob et al. 1999), and Perceived Stress Scale (PSS) (Cohen et al. 1983)—and indicated their level of pain and pruritus on a visual analogue scale (VAS).

### Intervention

The participants received psoriasis spa therapy in the establishment of their choice among the five French spa therapy centers participating in the study: La Roche Posay, Uriage, Saint-Gervais, Avène, and Molitg-Les-Bains. The composition of the natural thermal mineral waters differed in their composition from one center to another.

The immediate spa therapy group (hereafter called the “intervention group”) was requested to attend a 3-week course of spa therapy at focused on dermatology within 45 days after inclusion, while the control group received usual patient care until the visit at 4.5 months, then attended a 3-week course of dermatology-oriented spa therapy.

Procedures during the spa therapy were the subject of a prior consensus between the 5 participating thermal spa centers. The 18-day dermatology-oriented course of spa therapy was centered around the following 4 treatments (carried out daily): a filiform shower (water pressure of 4 to 15 bars), followed by balneotherapy in a pool (simple or bubbling bath for 20 min), full body and facial sprays (5 to 10 min), and localized treatment (bath, spray, showers, etc.) depending on the center. In addition to these treatments, participants attended two workshops per week: “relaxation or sophrology” and “hygiene and hydration.”

### Outcome measures assessments

The primary outcome measure was QoL specific to dermatology assessed through the DLQI score at 4.5-month post-randomization. In brief, DLQI evaluates the overall impact of skin disease on patients. It has 10 questions; the total score can range from 0 (no repercussions) to 30 (significant deterioration in quality of life) and 10 is the threshold at which there is a very large effect on QoL.^12^ Success was defined as a DLQI score at 4.5 months of ≤ 10.

The secondary outcome measures were as follows: change in the QoL specific to dermatology assessed by the evolution of the DLQI score; the proportion of patients with a DLQI ≤ 5 and the proportion of patients with a French-validated VQ-Dermato score (Grob et al. 1999^)^ ≤ 35; any improvement in overall QoL assessed using the EQ5D-3L questionnaire; effect clinical improvement in psoriasis, assessed using the PASI score; evaluation of pain and pruritus via VAS completed by the patient. To evaluate patients’ medical care, we collected psoriasis treatments, hospitalizations, specialist consultations, and the evolution of overall metabolism indicators by measuring waist circumference, body mass index (BMI), and blood pressure; and an evaluation of the side effects of spa therapy according to the usual pharmacovigilance criteria. Investigators evaluated all these secondary endpoints at the inclusion, 4.5-, 6-, 9-, and 12-month visits.

### Sample size calculation

The initial hypothesis was a proportion of patients with DLQI ≤ 10 of 25% at 4.5 months in the intervention (immediate spa therapy) group versus 10% in the control-delayed group. We thus planned to include 130 patients per group, or 260 in total, with an alpha risk of 0.05 and power of 80%.

Given the uncertainties relating to these hypotheses, the protocol provided for a reassessment of the number of subjects required after the first 100 inclusions, without an intermediate analysis. The success rate observed when reassessing the number of subjects required was 54% (both groups combined). With an alpha of 5% and a power of 80%, the number of subjects required would thus have been 49 patients per group, or 98 in total. When this number of patients was reached, it was nevertheless decided to continue inclusions until the end of the spa season to take into account of patients being lost to follow-up.

### Statistical analysis

Statistical analysis was in intention to treat. We present qualitative variables using number and frequency; and mean and standard deviation; or median and IQR (25th and 75th percentiles) for continuous variables, according to the distribution of the data, as well as the minimum and maximum values. We compared the success rate in the two groups using a Chi2 test. The relative risk (RR) of success with its 95% confidence interval (95% CI), the number needed to treat (NNT), and the effect size are also presented.

To analyze the change over time (M0–M4.5) of the quantitative secondary endpoints, we used a mixed model with analysis of the time*treatment interaction.

For qualitative secondary endpoints, we use the Chi2 test to compare the two groups at 4.5 months (if the conditions of application were met, otherwise Fisher’s exact test).

The statistical tests are carried out with a type one risk of error (alpha) of 5%.

Statistical analysis was performed after database lock using Stata 15.0 software (StataCorp, College Station, Texas).

More details can be found in the online supplementary material.

## Results

### Patients

Between January 2015 and November 2018, 128 patients were included in the study and randomized: 66 to the intervention group (immediate spa therapy) and 62 to the control group (delayed spa therapy). The last follow-up visit was in December 2019.

**Fig. 1:**
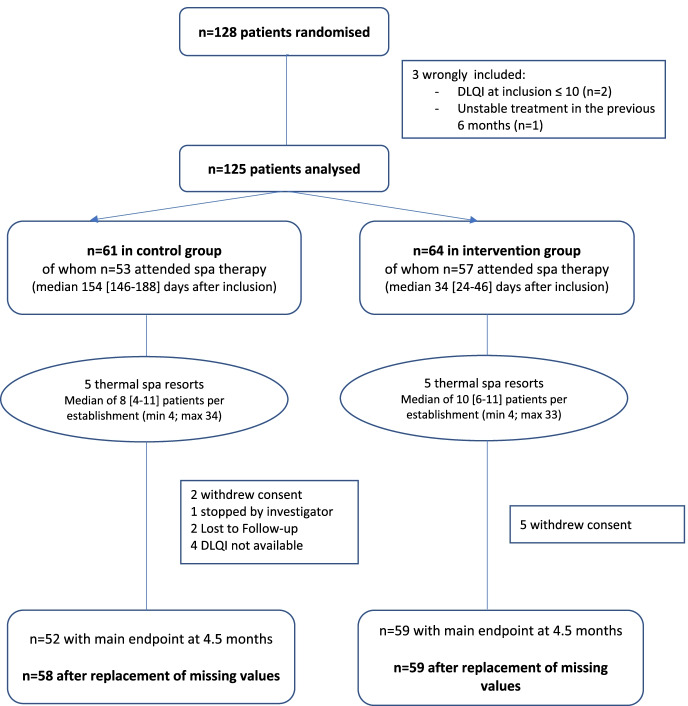
Study flow chart

In the intervention group, two patients were erroneously included (instability of treatment in the last 6 months and DLQI score at inclusion ≤ 10) and five withdrew their consent. The primary endpoint was therefore available for only 59 patients. In the control group, one patient was erroneously included (DLQI at inclusion ≤ 10), two withdrew their consent, one was withdrawn by the investigator, two were lost to follow-up, and for 4, no DLQI score was available at 4.5 months. The primary endpoint was finally available for 52 patients in the control group (Fig. 1).

Table 1 resumes the characteristics of the patients. DLQI and PASI scores were comparable in the two study groups: mean DLQI = 16.7 (range 11 to 30) and mean PASI = 10.5 (range: 0.7 to 50.1). VQ-Dermato sub-dimensions at inclusion in the study are presented in Supplementary Table S1.

**Table 1:** Main characteristics of patients at inclusion in the study

	Intervention group (*n* = 64)	Control group (*n* = 61)	Whole study population (*n* = 125)
Age, mean (SD) (min–max)	52.8 (15.3) (23–85)	51.4 (12.8) (21–74)	52.1 (14.1) (21–85)
Sex (male), *n* (%)	37 (57.8)	39 (63.9)	76 (60.8)
BMI, mean (SD) (min–max)	27.2 (5.0) (18.3–38.1)	27.4 (5.0) (17.1–40.0)	27.3 (5.0) (17.1–40.0)
Waist circumference, mean (SD) (min–max)	97.1 (17.3) (62–143) *(n* = *62)*	96.8 (14.9) (68–125) *(n* = *54)*	96.9 (16.2) (62–143) *(n* = *116)*
Thermal Spa resort, *n* (%)			
1. St Gervais	10 (15.6)	8 (13.1)	18 (14.4)
2. La Roche Posay	33 (51.6)	34 (55.7)	67 (53.6)
3. Molitg	4 (6.2)	4 (6.6)	8 (6.4)
4. Avène	6 (9.4)	4 (6.6)	10 (8.0)
5. Uriage	11 (17.2)	11 (18.0)	22 (17.6)
First-time spa curist, *n* (%)	43 (67.2)	46 (75.4)	89 (71.2)
Ongoing psoriasis treatments, *n* (%) *(several replies possible)*			
Corticosteroids	22 (34.4)	26 (42.6)	48 (38.4)
Vitamin D derivatives	25 (39.1)	23 (37.7)	48 (38.4)
Methotrexate	4 (6.2)	6 (9.8)	10 (8.0)
DLQI, mean (SD) (min–max)	16.6 (4.4) (11–29)	16.8 (5.4) (11–30)	16.7 (4.9) (11–30)
DLQI bands, *n* (%)			
[11–15]	32 (50.0)	31 (50.8)	63 (50.4)
[16–30]	32 (50.0)	30 (49.2)	62 (49.6)
PASI, mean (SD) (min–max)	10.2 (8.1) (0.8–38.7) *(n* = *63)*	10.8 (7.9) (0.7–50.1) *(n* = *61)*	10.5 (8.0) (0.7–50.1) *(n* = *124)*
VQ-Dermato, mean (SD) (min–max)	67.2 (11.6) (37.8–90.1) *(n* = *59)*	65.2 (16.1) (25.5–92.8) *(n* = *54)*	66.3 (13.9) (25.5–92.8) *(n* = *113)*
EQ5D-3L index, mean (SD) (min–max)	0.57 (0.27) (− 0.17–1)	0.61 (0.29) (− 0.09–1)	0.59 (0.28) (− 0.17–1)
PSS scale 14, mean (SD) (min–max)	27.8 (7.1) (8–41)	27.2 (6.9) (9–40)	27.5 (7.0) (8–41)
Pain VAS, mean (SD) (min–max)	4.1 (2.8) (0–10)	3.7 (3.1) (0–10)	3.9 (3.0) (0–10)
Pruritus VAS, mean (SD) (min–max)	6.7 (2.4) (1.1–10)	6.8 (2.5) (0–10)	6.8 (2.5) (0–10)

### Main endpoint

The success rate at 4.5 months, i.e., number of patients with a DLQI score at 4.5 months of ≤ 10, was 39/59 (66.1%) in the intervention group versus 22/52 (42.3%) in the control group, with a statistically significant difference (*p* = 0.012), before replacement of missing values. The result was similar after the replacement of missing values, with again significantly higher success in the intervention group compared to the control group (39/59 (66.1%) vs 24/58 (41.4%); *p* = 0.007) (Table 2). The RR of success was 1.60 (1.12–2.28) and the NNT was four patients (after replacement of missing values).

**Table 2:** Primary and secondary endpoints

		Intervention group	Control croup	*p* value
Primary endpoint				
DLQI ≤ 10 at 4.5 months^a^, *n*/*N* (%)		39/59 (66.1)	24/58 (41.4)	0.007
Quantitative secondary endpoints	Month			
VQ-Dermato, mean (SD)	0	67.2 (11.6) *n* = 59	65.2 (16.1) *n* = 54	0.003
	4.5	44.8 (20.6) *n* = 55	56.1 (20.1) *n* = 45	
Self-image, mean (SD)	0	57.1 (19.9) *n* = 64	57.8 (23.8) *n* = 60	0.010
	4.5	39.6 (26.3) *n* = 58	50.8 (26.0) *n* = 52	
Daily activities, mean (SD)	0	50.5 (20.9) *n* = 64	45.1 (23.6) *n* = 60	0.073
	4.5	34.1 (26.2) *n* = 58	35.6 (25.6) *n* = 52	
Mood, mean (SD)	0	64.6 (19.5) *n* = 63	62.6 (22.8) *n* = 61	0.084
	4.5	47.7 (24.0) *n* = 59	53.4 (28.6) *n* = 52	
Social Life, mean (SD)	0	57.7 (17.3) *n* = 63	59.3 (20.7) *n* = 61	0.017
	4.5	34.1 (26.4) *n* = 58	46.9 (26.4) *n* = 51	
Leisure activities, mean (SD)	0	70.1 (22.6) *n* = 64	72.1 (20.3) *n* = 61	0.071
	4.5	47.0 (28.7) *n* = 58	59.7 (25.8) *n* = 51	
Restricted due to treatment, mean (SD)	0	72.2 (21.9) *n* = 59	67.3 (29.8) *n* = 55	0.012
	4.5	54.1 (27.6) *n* = 58	66.3 (28.0) *n* = 46	
Physical discomfort, mean (SD)	0	81.2 (18.9) *n* = 64	79.1 (25.3) *n* = 61	< 0.01
	4.5	56.1 (25.4) *n* = 59	72.8 (23.1) *n* = 52	
Pruritus VAS, mean (SD)	0	6.7 (2.4) *n* = 64	6.8 (2.5) *n* = 61	0.047
	4.5	4.3 (2.7) *n* = 59	5.4 (2.8) *n* = 50	
Pain VAS, mean (SD)	0	4.1 (2.8) *n* = 64	3.7 (3.1) *n* = 61	0.309
	4.5	2.9 (2.8) *n* = 59	3.0 (2.9) *n* = 50	
EQ5D-3L index, mean (SD)	0	0.57 (0.27) *n* = 64	0.61 (0.29) *n* = 61	0.191
	4.5	0.69 (0.27) *n* = 59	0.66 (0.30) *n* = 51	
EQ5D-3L perceived state of health, mean (SD)	0	52.9 (18.3) *n* = 64	54.8 (20.8) *n* = 60	0.538
	4.5	60.5 (20.6) *n* = 58	65.5 (20.3) *n* = 51	
Perceived stress: PSS 14, mean (SD)	0	27.8 (7.1) *n* = 64	27.2 (6.9) *n* = 61	0.498
	4.5	24.9 (7.1) *n* = 59	25.4 (9.0) *n* = 52	
Qualitative secondary endpoints				
VQ-Dermato ≤ 35 at 4.5 months, *n*/*N* (%)		17/55 (30.9)	8/45 (17.8)	0.131
PASI50 at 4.5 months, *n*/*N* (%)		14/56 (25.0)	14/53 (26.4)	0.866
PASI75 at 4.5 months, *n*/*N* (%)		8/56 (14.3)	5/53 (9.4)	0.435

^a^After replacement of missing values

*DLQI*, Dermatology Life Quality Index; *EQ5D-3L*, health-related quality of life score

### Secondary endpoints

**Fig. 2:**
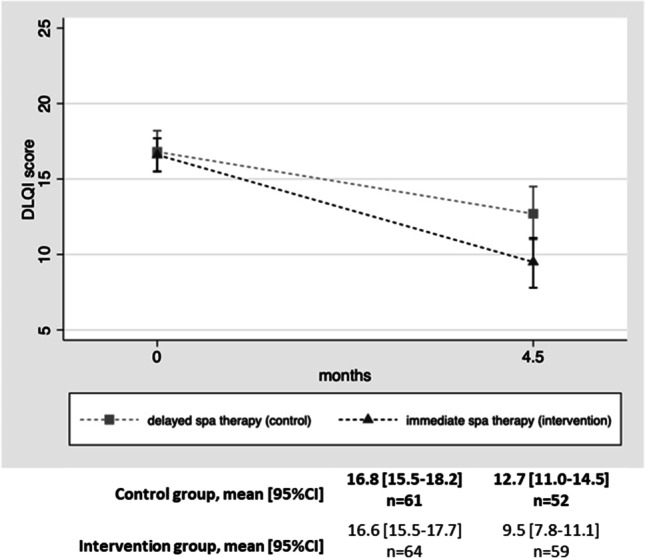
Change in DLQI score with time

The change in DLQI between inclusion and 4.5 months was statistically different between the two groups (*p* = 0.012) (Fig. 2). The effect size was 0.61. The proportion of patients achieving a DLQI ≤ 5 at 4.5 months was 15/59 (25.4%) in the intervention group versus 6/52 (11.5%) in the control group (*p* = 0.062). The proportion of patients with a decrease of at least 5 points in their DLQI score at 4.5 months (compared with their score at inclusion) was 43/59 (72.9%) in the intervention group versus 24/52 (46.2%) in the control group (*p* = 0.004).

Table 2 presents the secondary endpoints. The evolution of VQ-Dermato between inclusion and 4.5 months was statistically different between the two groups (*p* = 0.003), in particular for the sub-dimensions assessing self-image, social life, limitations due to treatment, and physical discomfort. The VAS for pruritus fell from 6.7 (± 2.4) at inclusion to 4.3 (± 2.7) at 4.5 months in the intervention group, whereas it decreased from 6.8 (± 2.5) at inclusion to 5.4 (± 2.8) at 4.5 months in the control group with a statistically significant difference between the two groups (*p* = 0.047). Conversely, no significant difference was found concerning the VAS pain estimation, the overall quality of life measured by the EQ5D-3L questionnaire, the clinical evaluation of psoriasis measured through the PASI, or stress evaluated using the PSS scale.

The consumption of medications at 4.5 months was not statistically different between the two groups, nor was the consumption of care and consultations, whether or not related to psoriasis (Supplementary Table S2).

We observed no impact of spa therapy on the patient’s metabolism. The change between inclusion and 4.5 months of the various parameters (BMI, waist circumference, PAS, PAD, and heart rate) was not statistically different between the two groups (data not shown).

### Long-term assessment: outcome 12 months after completion of spa treatment

**Fig. 3:**
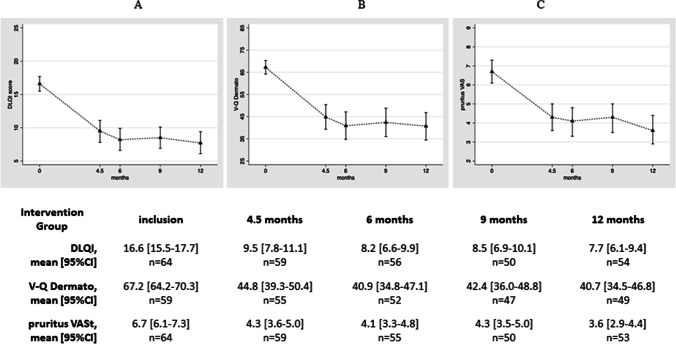
Long-term assessment in the intervention (immediate spa therapy) group

For the intervention group, we observed long-term stability of the results for the DLQI with maintenance of benefits up to 12 months. This was similar for the VQ-Dermato score and the VAS estimate of pruritus (Fig. 3).

### Safety

Nine SAEs, all consisting in unexpected hospitalizations, were reported during the study: four in the intervention group (worsening of psoriasis, flare-up of pustular erythrodermic psoriasis, an acute psychotic disorder, and thromboendarteriectomy) and five in the control group (cholecystectomy, glioblastoma, hysteroscopy, acute and severe facial eczema, and myocardial infarction with implantation of a stent).

### Planned subgroup analysis

The result obtained for the primary endpoint was not different whatever the spa establishment, the severity of psoriasis at baseline (DLQI band at inclusion 11–15 versus 16–30), or whether the participant had received spa therapy previously (Mantel–Haenszel, OR, and homogeneity test, *p* = 0.954, *p* = 0.391, and *p* = 0.198, respectively).

### Adherence to therapy

Participants in the intervention group received the majority of the treatments planned during the spa therapy course (Table 3).

**Table 3:** Spa treatments received by the patients in the intervention group

	Intervention group (*n* = 54)
Filiform shower at variable pressure	
At least one, *n* (%)	54/54 (100)
Number, median [IQR] (min–max)	15 [12–15] (5–15) *(n* = *54)*
Pressure used ≥ 15 bars, *n*/*N* (%)	37/51 (72.5%)
Aerobath	
At least one, *n* (%)	45/54 (83.3)
Number, median [IQR] (min–max)	18 [18–18] (13–18) *(n* = *45)*
Whole body sprays	
At least one, *n* (%)	54/54 (100)
Number, median [IQR] (min–max)	18 [16–18] (11–36) *(n* = *54)*
Localized treatments (depending on the resort)^b^	
At least one, *n* (%)	54/54 (100)
Number, median [IQR] (min–max)	18 [15–18] (11–18) *(n* = *54)*
Hygiene/hydration workshop	
At least one, *n* (%)	33/54 (61.1)
Relaxation/sophrology workshop	
At least one, *n* (%)	34/54 (63.0)

^b^Mainly facial spray and scalp care

## Discussion

To date, to our knowledge, this is the first randomized controlled trial that evaluated spa therapy for psoriasis. To limit any disappointment bias, all patients were proposed a course of spa therapy, albeit at different times from inclusion, i.e., the control group was offered a course of spa therapy after the assessment of the primary endpoint at 4.5 months after inclusion in the trial.

We demonstrated that a cure of spa therapy improved QoL and alleviated certain symptoms of psoriasis, in both the short and long term. Furthermore, we managed to collect the details about all the procedures provided during the 3-week course of spa therapy along with the participants’ adherence to these treatments, which was excellent. We observed no center effect in this multicenter study, possibly thanks to the standardization of treatments agreed by consensus prior to the trial. This represents a strength for our results, as this suggests that they might be generalized to other spa centers/resorts using the same protocol.

With our main criterion of success achieved—DLQI ≤ 10, 4.5 months after inclusion with an encouraging effect size (NNT = 4)—we demonstrated that spa treatment leads to an improvement in QoL specific to skin disease as assessed by this score. Our main criterion of success was attaining a DLQI score ≤ 10. Therefore, our results cannot be compared to those obtained with systemic treatments where the efficacy assessment is based on the PASI score and where the DLQI evaluations consider DLQI ≤ 5 or 0/1 (Nast et al. 2020). However, such comparison is not really relevant as spa therapy does not represent an alternative to any treatment but is rather an add-on or complementary patient-centered approach (Matz et al. 2003).

Our secondary criterion (VQ-Dermato) also shows an improvement in the dermatological aspects of QoL in particular facets such as self-image, social life, limitations due to treatment, and physical discomfort.

Pruritus affects about 70% of patients with psoriasis and it is the most bothersome symptom for them (Szepietowski and Reich 2016). We showed that the intervention relieves this major symptom, with a significant improvement in the subjective VAS pruritus score that may be directly due to the quality of the thermal spa water. This is in contrast to the effect observed in a study at the Salies de Béarn resort, where saline water had an irritating effect with pruritus and a sensation of burning (Léauté-Labrèze et al. 2001).

Although not significant and only moderate, an improved objective PASI75 score was obtained in 14.3% of patients in the intervention group and 9.4% in the control group.

Of particular interest was the significant medium-term result of our study which was maintained in the long-term evaluation at 12 months for DLQI, VQ-Dermato, and pruritus. This result is not commonly reported in studies evaluating spa therapy where the effects are lost within a few months (Peroni et al. 2008).

Our study mainly included patients naive to spa therapy. We detected no interaction bias between naive patients and those who had previously received spa therapy. We found no effect of disease severity, suggesting a benefit whatever the degree of severity. While this prevents us from identifying the best candidates for spa therapy, it suggests that spa therapy may be proposed to all patients, if their psoriasis is stable, especially those with stable psoriasis and a high DLQI score.

A possible limitation was the modest size of the study, albeit in line with the planned sample size. Studies with interventions lasting several weeks often have difficulties in recruiting eligible patients of working age. Another limitation was the absence of detailed information on the composition of the mineral waters at each of the study centers. This, together with the relatively small study population, meant that we were unable to make an analysis in terms of the properties of the mineral waters.

Finally, this was a pragmatic study evaluating a combination of balneotherapy, physiotherapy in a pool, well-being cares, rest, and patient workshops taken together, without it being possible to detail the effect of one or the other treatment, in particular the filiform shower administered by a thermal spa dermatologist.

Our results justify integrating spa therapy into the treatments offered to patients with psoriasis, whether they have other ongoing treatment or not. However, the profile of patients who would most benefit from a course of spa treatment remains to be determined.

## Supplementary Information

Below is the link to the electronic supplementary material.

Supplementary file1 (DOCX 25 KB)

## Data Availability

Anonymized study data will be made available following publication on reasonable request to the corresponding author.

## Abbreviations

BMI: Body mass index
DLQI: Dermatology Life Quality Index
GrPso: French Psoriasis Research Group
NNT: Number needed to treat
OR: Odds ratio
PASI: Psoriasis Area and Severity Index
PSS: Perceived Stress Scale
QoL: Quality of life
RR: Relative risk
UVB: Ultraviolet B
VAS: Visual analogue scale
