# Supplementary material for: Evaluation of the benefit of thermal spa therapy in plaque psoriasis: the PSOTHERMES randomized clinical trial
Source: Int J Biometeorol. 2022 Mar 26;66(6):1247–56. doi: 10.1007/s00484-022-02273-7 (PMC9132808; doi:10.1007/s00484-022-02273-7)
Supplement: Supplementary file 1 — Supplementary file1 (DOCX 25 KB) [file 484_2022_2273_MOESM1_ESM.docx]

**Evaluation of the benefit of thermal spa treatment in plaque psoriasis: the PSOTHERMES randomized clinical trial**

M. Beylot-Barry^1,2^, E.Mahé^2,3^, C. Rolland MSc^4^, M. Amy de la Bretèque^3^, Claire Eychenne^4^, J. Charles^2,5^, C. Payen^6^, L Machet^7^, C. Vermorel^4^, A. Foote^8^, C. Roques^9^, J-L. Bosson^4^

1-Department of Dermatology, University Hospital of Bordeaux, France

2- French Psoriasis Research Group (GrPSO) of the French Society of Dermatology

3-Department of Dermatology, Victor Dupouy Hospital, Argenteuil, France

4-CNRS TIMC-Imag Laboratory, University Grenoble Alpes, France

5-Department of Dermatology, University Hospital of Grenoble, and INSERM U1209 University Grenoble Alpes, France

6-Private practice, Place Louis Jouvet, Grenoble, France

7-Department of Dermatology, University Hospital of Tours, France

8-Research Division, Grenoble Alpes University Hospital, Grenoble, France

9-French Association for Thermal Research (AFRETH), Paris, France

**Corresponding author**: **Prof.Jean-Luc Bosson,** TIMC-IMAG Laboratory, Pavillon Taillefer CHU Grenoble-Alpes, CS10217 38043 Grenoble, France

Email: [jean-luc.bosson@univ-grenoble-alpes.fr](mailto:jean-luc.bosson@univ-grenoble-alpes.fr)
Tel: 33(0)476765047

ORCID: 0000-0003-0967-6026

**Supplementary material**

**Further details on statistical methods used**

For the main endpoint, missing values were replaced using multivariate logistic regression to search for factors associated with success, integrating several explanatory variables (group, DLQI at inclusion, age, sex, first-time patient, PASI at inclusion). Patients with a model probability> 0.5 were classified as successes and patients with a model probability ≤ 0.5 as failures. For patients who withdrew consent, or were withdrawn by the investigator, data on the primary endpoint was not replaced.

The effect size between the groups was calculated by: mean of the difference M4.5-M0 in the intervention group minus mean of the difference M4.5-M0 in the control group divided by standard deviation at M0 for the two groups combined.

We used the Mantel-Haenszel OR homogeneity test according to the stratification criteria, on the primary endpoint.

For long-term follow-up in the intervention group, we used a mixed model with time effect analysis (M0-M4.5-M6-M9-M12).

**Supplementary Results**

**Table S1: V-Q Dermato sub-dimensions at inclusion in the study**

| V-Q Dermato sub-dimensions | **Intervention group**  (n=64) | **Control group**  (n=61) | Whole study population (n=125) |
| --- | --- | --- | --- |
| Self-image, mean (SD) (min-max) | 57.1 (19.9)  (14.3-100)  *(n=64)* | 57.8 (23.8)  (7.1-100)  *(n=60)* | 57.4 (21.8)  (7.1-100)  *(n=124)* |
| Daily activities, mean (SD) (min-max) | 50.5 (20.9)  (5.0-100)  *(n=64)* | 45.1 (23.6)  (5.0-95.0)  *(n=60)* | 47.9 (22.3)  (5.0-100)  *(n=124)* |
| Mood, mean (SD) (min-max) | 64.6 (19.5)  (6.3-100)  *(n=63)* | 62.6 (22.8)  (6.3-100)  *(n=61)* | 63.6 (21.2)  (6.3-100)  *(n=124)* |
| Social Life, mean (SD) (min-max) | 57.7 (17.3)  (10.0-100)  *(n=63)* | 59.3 (20.7)  (15.0-95.0)  *(n=61)* | 58.5 (19.0)  (10.0-100)  *(n=124)* |
| Leisure activities, mean (SD) (min-max) | 70.1 (22.6)  (8.3-100) | 72.1 (20.3)  (16.7-100) | 71.0 (21.4)  (8.3-100) |
| Restricted due to treatment, mean (SD) (min-max) | 72.2 (21.9)  (12.5-100)  *(n=59)* | 67.3 (29.8)  (0-100)  *(n=55)* | 69.8 (26.0)  (0-100)  *(n=114)* |
| Physical discomfort, mean (SD) (min-max) | 81.2 (18.9)  (25.0-100) | 79.1 (25.3)  (0-100) | 80.2 (22.2)  (0-100) |

**Table S2 : Ongoing treatments**

|  | **Intervention Group** | **Control Group** | p-value |
| --- | --- | --- | --- |
| **Medication consumption at 4.5 months** | n=57 | n=52 |  |
| Corticosteroids, n (%) | 21 (36.8) | 24 (46.2) | 0.32 |
| Vitamin D derivatives, n (%) | 22 (38.6) | 17 (32.7) | 0.52 |
| Methotrexate, n (%) | 6 (10.5) | 7 (13.5) | 0.64 |
| **Healthcare consumption between inclusion and 4.5 months** |  |  |  |
| Topical treatments (tubes per month, n (%) | n=57 | n=51 |  |
| 0 | 19 (33.3) | 20 (39.2) | 0.49 |
| 1 | 23 (40.4) | 15 (29.4) |  |
| 2+ | 15 (26.3) | 16 (31.4) |  |
| Number of phototherapy sessions, n (%) | n=57 | n=51 |  |
| none | 56 (98.2) | 49 (96.1) | 0.60 |
| at least 1 | 1 (1.8) | 2 (3.9) |  |
| Use of conventional systemic treatments, n (%) | n=56 | n=51 |  |
| no | 53 (94.6) | 43 (84.3) | 0.08 |
| yes | 3 (5.4) | 8 (15.7) |  |
| Weeks of biotherapy treatment, n (%) | n=55 | n=51 |  |
| none | 54 (98.2) | 49 (96.1) | 0.61 |
| at least 1 | 1 (1.8) | 2 (3.9) |  |
| **Consultations related to psoriasis between inclusion and 4.5 months** | n=57 | n=52 |  |
| None , n (%) | 42 (73.7) | 42 (80.8) | 0.50 |
| 1 | 10 (17.5) | 5 (9.6) |  |
| 2+ | 5 (8.8) | 5 (9.6) |  |
| **Consultations unrelated to psoriasis between inclusion and 4.5 months** | n=57 | n=52 |  |
| None, n (%) | 40 (70.2) | 36 (69.2) | 0.88 |
| 1 | 12 (21.1) | 10 (19.2) |  |
| 2+ | 5 (8.8) | 6 (11.5) |  |
